# Supplementary material for: Resolving the full spectrum of human genome variation using Linked-Reads
Source: Genome Res. 2019 Apr;29(4):635–45. doi: 10.1101/gr.234443.118 (PMC6442396; doi:10.1101/gr.234443.118)
Supplement: Supplemental Material [file supp_gr.234443.118_Supplemental_longranger-2.2.zip › longranger-2.2.tar.bz2!/lr2.2-public/lib/stan/cmdstan/stan/doc/api/html/model_2util_8hpp_source.html]

Stan: src/stan/model/util.hpp Source File


|  |  |
| --- | --- |
|  | Stan  2.5.0  probability, sampling & optimization |


- Main Page
- Related Pages
- Namespaces
- Classes
- Files

- File List
- File Members

All Classes Namespaces Files Functions Variables Typedefs Enumerations Enumerator Friends Macros Pages

- src
- stan
- model

util.hpp

Go to the documentation of this file.

1 #ifndef STAN\_\_MODEL\_\_UTIL\_HPP

2 #define STAN\_\_MODEL\_\_UTIL\_HPP

3

4 #include <cmath>

5 #include <iomanip>

6 #include <iostream>

7 #include <vector>

8

9 #include <stan/math/matrix/Eigen.hpp>

10 #include <stan/agrad/rev/matrix/grad.hpp>

11 #include <stan/agrad/rev/var.hpp>

12 #include <stan/agrad/rev/var.hpp>

13 #include <stan/agrad/autodiff.hpp>

14

15 namespace stan {

16

17  namespace model {

18

40  template <bool jacobian\_adjust\_transform, class M>

41  double log\_prob\_propto(const M& model,

42  std::vector<double>& params\_r,

43  std::vector<int>& params\_i,

44  std::ostream\* msgs = 0) {

45  using stan::agrad::var;

46  using std::vector;

47  vector<var> ad\_params\_r;

48  for (size\_t i = 0; i < model.num\_params\_r(); ++i)

49  ad\_params\_r.push\_back(params\_r[i]);

50  try {

51  double lp

52  = model

53  .template log\_prob<true,

54  jacobian\_adjust\_transform>(ad\_params\_r,params\_i,

55  msgs)

56  .val();

57  stan::agrad::recover\_memory();

58  return lp;

59  } catch (std::exception &ex) {

60  stan::agrad::recover\_memory();

61  throw;

62  }

63  }

64

82  template <bool propto, bool jacobian\_adjust\_transform, class M>

83  double log\_prob\_grad(const M& model,

84  std::vector<double>& params\_r,

85  std::vector<int>& params\_i,

86  std::vector<double>& gradient,

87  std::ostream\* msgs = 0) {

88  using std::vector;

89  using stan::agrad::var;

90  double lp;

91  try {

92  vector<var> ad\_params\_r(params\_r.size());

93  for (size\_t i = 0; i < model.num\_params\_r(); ++i) {

94  stan::agrad::var var\_i(params\_r[i]);

95  ad\_params\_r[i] = var\_i;

96  }

97  var adLogProb

98  = model

99  .template log\_prob<propto,

100  jacobian\_adjust\_transform>(ad\_params\_r,

101  params\_i,msgs);

102  lp = adLogProb.val();

103  adLogProb.grad(ad\_params\_r,gradient);

104  } catch (const std::exception &ex) {

105  stan::agrad::recover\_memory();

106  throw;

107  }

108  stan::agrad::recover\_memory();

109  return lp;

110  }

111

132  template <bool jacobian\_adjust\_transform, class M>

133  double log\_prob\_propto(const M& model,

134  Eigen::VectorXd& params\_r,

135  std::ostream\* msgs = 0) {

136  using stan::agrad::var;

137  using std::vector;

138  double lp;

139  try {

140  vector<var> ad\_params\_r;

141  for (size\_t i = 0; i < model.num\_params\_r(); ++i)

142  ad\_params\_r.push\_back(params\_r(i));

143  lp

144  = model

145  .template log\_prob<true,

146  jacobian\_adjust\_transform>(ad\_params\_r, msgs)

147  .val();

148  } catch (std::exception &ex) {

149  stan::agrad::recover\_memory();

150  throw;

151  }

152  stan::agrad::recover\_memory();

153  return lp;

154  }

155

172  template <bool propto, bool jacobian\_adjust\_transform, class M>

173  double log\_prob\_grad(const M& model,

174  Eigen::VectorXd& params\_r,

175  Eigen::VectorXd& gradient,

176  std::ostream\* msgs = 0) {

177  using std::vector;

178  using stan::agrad::var;

179  Eigen::Matrix<var,Eigen::Dynamic,1> ad\_params\_r(params\_r.size());

180  for (size\_t i = 0; i < model.num\_params\_r(); ++i) {

181  stan::agrad::var var\_i(params\_r[i]);

182  ad\_params\_r[i] = var\_i;

183  }

184  try {

185  var adLogProb

186  = model

187  .template log\_prob<propto,

188  jacobian\_adjust\_transform>(ad\_params\_r, msgs);

189  double val = adLogProb.val();

190  stan::agrad::grad(adLogProb, ad\_params\_r, gradient);

191  return val;

192  } catch (std::exception &ex) {

193  stan::agrad::recover\_memory();

194  throw;

195  }

196  }

197

216  template <bool propto, bool jacobian\_adjust\_transform, class M>

217  void finite\_diff\_grad(const M& model,

218  std::vector<double>& params\_r,

219  std::vector<int>& params\_i,

220  std::vector<double>& grad,

221  double epsilon = 1e-6,

222  std::ostream\* msgs = 0) {

223  std::vector<double> perturbed(params\_r);

224  grad.resize(params\_r.size());

225  for (size\_t k = 0; k < params\_r.size(); k++) {

226  perturbed[k] += epsilon;

227  double logp\_plus

228  = model

229  .template log\_prob<propto,

230  jacobian\_adjust\_transform>(perturbed, params\_i,

231  msgs);

232  perturbed[k] = params\_r[k] - epsilon;

233  double logp\_minus

234  = model

235  .template log\_prob<propto,

236  jacobian\_adjust\_transform>(perturbed, params\_i,

237  msgs);

238  double gradest = (logp\_plus - logp\_minus) / (2\*epsilon);

239  grad[k] = gradest;

240  perturbed[k] = params\_r[k];

241  }

242  }

243

244

267  template <bool propto, bool jacobian\_adjust\_transform, class M>

268  int test\_gradients(const M& model,

269  std::vector<double>& params\_r,

270  std::vector<int>& params\_i,

271  double epsilon = 1e-6,

272  double error = 1e-6,

273  std::ostream& o = std::cout,

274  std::ostream\* msgs = 0) {

275  std::vector<double> grad;

276  double lp

277  = stan::model::log\_prob\_grad<propto,

278  jacobian\_adjust\_transform>(model,

279  params\_r,

280  params\_i,

281  grad,msgs);

282

283  std::vector<double> grad\_fd;

284  finite\_diff\_grad<false,

285  true,

286  M>(model,

287  params\_r, params\_i,

288  grad\_fd, epsilon,

289  msgs);

290

291  int num\_failed = 0;

292

293  o << std::endl

294  << " Log probability=" << lp

295  << std::endl;

296

297  o << std::endl

298  << std::setw(10) << "param idx"

299  << std::setw(16) << "value"

300  << std::setw(16) << "model"

301  << std::setw(16) << "finite diff"

302  << std::setw(16) << "error"

303  << std::endl;

304  for (size\_t k = 0; k < params\_r.size(); k++) {

305  o << std::setw(10) << k

306  << std::setw(16) << params\_r[k]

307  << std::setw(16) << grad[k]

308  << std::setw(16) << grad\_fd[k]

309  << std::setw(16) << (grad[k] - grad\_fd[k])

310  << std::endl;

311  if (std::fabs(grad[k] - grad\_fd[k]) > error)

312  num\_failed++;

313  }

314  return num\_failed;

315  }

316

317

340  template <bool propto, bool jacobian\_adjust\_transform, class M>

341  double grad\_hess\_log\_prob(const M& model,

342  std::vector<double>& params\_r,

343  std::vector<int>& params\_i,

344  std::vector<double>& gradient,

345  std::vector<double>& hessian,

346  std::ostream\* msgs = 0) {

347  const double epsilon = 1e-3;

348  const int order = 4;

349  const double perturbations[order]

350  = {-2\*epsilon, -1\*epsilon, epsilon, 2\*epsilon};

351  const double coefficients[order]

352  = { 1.0 / 12.0,

353  -2.0 / 3.0,

354  2.0 / 3.0,

355  -1.0 / 12.0 };

356

357  double result

358  = stan::model::log\_prob\_grad<propto,

359  jacobian\_adjust\_transform>(model,

360  params\_r,

361  params\_i,

362  gradient,

363  msgs);

364  hessian.assign(params\_r.size() \* params\_r.size(), 0);

365  std::vector<double> temp\_grad(params\_r.size());

366  std::vector<double> perturbed\_params(params\_r.begin(), params\_r.end());

367  for (size\_t d = 0; d < params\_r.size(); d++) {

368  double\* row = &hessian[d\*params\_r.size()];

369  for (int i = 0; i < order; i++) {

370  perturbed\_params[d] = params\_r[d] + perturbations[i];

371  stan::model::log\_prob\_grad<propto,

372  jacobian\_adjust\_transform>(model,

373  perturbed\_params,

374  params\_i,

375  temp\_grad);

376  for (size\_t dd = 0; dd < params\_r.size(); dd++) {

377  row[dd] += 0.5 \* coefficients[i] \* temp\_grad[dd] / epsilon;

378  hessian[d + dd\*params\_r.size()]

379  += 0.5 \* coefficients[i] \* temp\_grad[dd] / epsilon;

380  }

381  }

382  perturbed\_params[d] = params\_r[d];

383  }

384  return result;

385  }

386

387  // Interface for automatic differentiation of models

388

389  template <class M>

390  struct model\_functional {

391

392  const M& model;

393  std::ostream\* o;

394

395  model\_functional(const M& m, std::ostream\* out): model(m), o(out) {};

396

397  template <typename T>

398  T operator()(Eigen::Matrix<T, Eigen::Dynamic, 1>& x) const {

399  return model.template log\_prob<true, true, T>(x, o);

400  }

401

402  };

403

404  template <class M>

405  void gradient(const M& model,

406  const Eigen::Matrix<double, Eigen::Dynamic, 1>& x,

407  double& f,

408  Eigen::Matrix<double, Eigen::Dynamic, 1>& grad\_f,

409  std::ostream\* msgs = 0) {

410

411  stan::agrad::gradient(model\_functional<M>(model, msgs), x, f, grad\_f);

412

413  }

414

415  template <class M>

416  void hessian(const M& model,

417  const Eigen::Matrix<double, Eigen::Dynamic, 1>& x,

418  double& f,

419  Eigen::Matrix<double, Eigen::Dynamic, 1>& grad\_f,

420  Eigen::Matrix<double, Eigen::Dynamic, Eigen::Dynamic>& hess\_f,

421  std::ostream\* msgs = 0) {

422

423  stan::agrad::hessian(model\_functional<M>(model, msgs), x, f, grad\_f, hess\_f);

424

425  }

426

427  template <class M>

428  void gradient\_dot\_vector(const M& model,

429  const Eigen::Matrix<double, Eigen::Dynamic, 1>& x,

430  const Eigen::Matrix<double, Eigen::Dynamic, 1>& v,

431  double& f,

432  double& grad\_f\_dot\_v,

433  std::ostream\* msgs = 0) {

434

435  stan::agrad::gradient\_dot\_vector(model\_functional<M>(model, msgs), x, v, f, grad\_f\_dot\_v);

436

437  }

438

439  template <class M>

440  void hessian\_times\_vector(const M& model,

441  const Eigen::Matrix<double, Eigen::Dynamic, 1>& x,

442  const Eigen::Matrix<double, Eigen::Dynamic, 1>& v,

443  double& f,

444  Eigen::Matrix<double, Eigen::Dynamic, 1>& hess\_f\_dot\_v,

445  std::ostream\* msgs = 0) {

446

447  stan::agrad::hessian\_times\_vector(model\_functional<M>(model, msgs), x, v, f, hess\_f\_dot\_v);

448

449  }

450

451  template <class M>

452  void grad\_tr\_mat\_times\_hessian(const M& model,

453  const Eigen::Matrix<double, Eigen::Dynamic, 1>& x,

454  const Eigen::Matrix<double, Eigen::Dynamic, Eigen::Dynamic>& X,

455  Eigen::Matrix<double, Eigen::Dynamic, 1>& grad\_tr\_X\_hess\_f,

456  std::ostream\* msgs = 0) {

457

458  stan::agrad::grad\_tr\_mat\_times\_hessian(model\_functional<M>(model, msgs), x, X, grad\_tr\_X\_hess\_f);

459

460  }

461

462

463  }

464 }

465

466

467

468 #endif

stan::agrad::gradient

void gradient(const F &f, const Eigen::Matrix< double, Eigen::Dynamic, 1 > &x, double &fx, Eigen::Matrix< double, Eigen::Dynamic, 1 > &grad\_fx)

Calculate the value and the gradient of the specified function at the specified argument.

**Definition:** autodiff.hpp:93

stan::agrad::fabs

fvar< T > fabs(const fvar< T > &x)

**Definition:** fabs.hpp:18

stan::agrad::recover\_memory

static void recover\_memory()

Recover memory used for all variables for reuse.

**Definition:** var\_stack.hpp:52

stan::model::log\_prob\_grad

double log\_prob\_grad(const M &model, std::vector< double > &params\_r, std::vector< int > &params\_i, std::vector< double > &gradient, std::ostream \*msgs=0)

Compute the gradient using reverse-mode automatic differentiation, writing the result into the specif...

**Definition:** util.hpp:83

stan::model::model\_functional

**Definition:** util.hpp:390

stan::math::row

Eigen::Matrix< T, 1, Eigen::Dynamic > row(const Eigen::Matrix< T, Eigen::Dynamic, Eigen::Dynamic > &m, size\_t i)

Return the specified row of the specified matrix, using start-at-1 indexing.

**Definition:** row.hpp:26

stan::model::hessian

void hessian(const M &model, const Eigen::Matrix< double, Eigen::Dynamic, 1 > &x, double &f, Eigen::Matrix< double, Eigen::Dynamic, 1 > &grad\_f, Eigen::Matrix< double, Eigen::Dynamic, Eigen::Dynamic > &hess\_f, std::ostream \*msgs=0)

**Definition:** util.hpp:416

stan::model::test\_gradients

int test\_gradients(const M &model, std::vector< double > &params\_r, std::vector< int > &params\_i, double epsilon=1e-6, double error=1e-6, std::ostream &o=std::cout, std::ostream \*msgs=0)

Test the log\_prob\_grad() function's ability to produce accurate gradients using finite differences...

**Definition:** util.hpp:268

grad.hpp

stan::agrad::var::val

double val() const

Return the value of this variable.

**Definition:** var.hpp:209

stan::agrad::grad

static void grad(chainable \*vi)

Compute the gradient for all variables starting from the specified root variable implementation.

**Definition:** chainable.hpp:110

stan::model::model\_functional::model

const M & model

**Definition:** util.hpp:392

stan::model::gradient

void gradient(const M &model, const Eigen::Matrix< double, Eigen::Dynamic, 1 > &x, double &f, Eigen::Matrix< double, Eigen::Dynamic, 1 > &grad\_f, std::ostream \*msgs=0)

**Definition:** util.hpp:405

stan::agrad::hessian\_times\_vector

void hessian\_times\_vector(const F &f, const Eigen::Matrix< double, Eigen::Dynamic, 1 > &x, const Eigen::Matrix< double, Eigen::Dynamic, 1 > &v, double &fx, Eigen::Matrix< double, Eigen::Dynamic, 1 > &Hv)

**Definition:** autodiff.hpp:274

stan::model::hessian\_times\_vector

void hessian\_times\_vector(const M &model, const Eigen::Matrix< double, Eigen::Dynamic, 1 > &x, const Eigen::Matrix< double, Eigen::Dynamic, 1 > &v, double &f, Eigen::Matrix< double, Eigen::Dynamic, 1 > &hess\_f\_dot\_v, std::ostream \*msgs=0)

**Definition:** util.hpp:440

stan::model::finite\_diff\_grad

void finite\_diff\_grad(const M &model, std::vector< double > &params\_r, std::vector< int > &params\_i, std::vector< double > &grad, double epsilon=1e-6, std::ostream \*msgs=0)

Compute the gradient using finite differences for the specified parameters, writing the result into t...

**Definition:** util.hpp:217

stan::model::model\_functional::model\_functional

model\_functional(const M &m, std::ostream \*out)

**Definition:** util.hpp:395

stan::agrad::grad\_tr\_mat\_times\_hessian

void grad\_tr\_mat\_times\_hessian(const F &f, const Eigen::Matrix< double, Eigen::Dynamic, 1 > &x, const Eigen::Matrix< double, Eigen::Dynamic, Eigen::Dynamic > &M, Eigen::Matrix< double, Eigen::Dynamic, 1 > &grad\_tr\_MH)

**Definition:** autodiff.hpp:321

stan::model::gradient\_dot\_vector

void gradient\_dot\_vector(const M &model, const Eigen::Matrix< double, Eigen::Dynamic, 1 > &x, const Eigen::Matrix< double, Eigen::Dynamic, 1 > &v, double &f, double &grad\_f\_dot\_v, std::ostream \*msgs=0)

**Definition:** util.hpp:428

stan::model::model\_functional::operator()

T operator()(Eigen::Matrix< T, Eigen::Dynamic, 1 > &x) const

**Definition:** util.hpp:398

var.hpp

stan::model::grad\_tr\_mat\_times\_hessian

void grad\_tr\_mat\_times\_hessian(const M &model, const Eigen::Matrix< double, Eigen::Dynamic, 1 > &x, const Eigen::Matrix< double, Eigen::Dynamic, Eigen::Dynamic > &X, Eigen::Matrix< double, Eigen::Dynamic, 1 > &grad\_tr\_X\_hess\_f, std::ostream \*msgs=0)

**Definition:** util.hpp:452

Eigen.hpp

stan::model::grad\_hess\_log\_prob

double grad\_hess\_log\_prob(const M &model, std::vector< double > &params\_r, std::vector< int > &params\_i, std::vector< double > &gradient, std::vector< double > &hessian, std::ostream \*msgs=0)

Evaluate the log-probability, its gradient, and its Hessian at params\_r.

**Definition:** util.hpp:341

stan::agrad::var

Independent (input) and dependent (output) variables for gradients.

**Definition:** var.hpp:27

stan::math::e

double e()

Return the base of the natural logarithm.

**Definition:** constants.hpp:86

stan::agrad::gradient\_dot\_vector

void gradient\_dot\_vector(const F &f, const Eigen::Matrix< T1, Eigen::Dynamic, 1 > &x, const Eigen::Matrix< T2, Eigen::Dynamic, 1 > &v, T1 &fx, T1 &grad\_fx\_dot\_v)

**Definition:** autodiff.hpp:252

stan::model::model\_functional::o

std::ostream \* o

**Definition:** util.hpp:393

autodiff.hpp

stan::model::log\_prob\_propto

double log\_prob\_propto(const M &model, std::vector< double > &params\_r, std::vector< int > &params\_i, std::ostream \*msgs=0)

Helper function to calculate log probability for double scalars up to a proportion.

**Definition:** util.hpp:41

stan::agrad::hessian

void hessian(const F &f, const Eigen::Matrix< double, Eigen::Dynamic, 1 > &x, double &fx, Eigen::Matrix< double, Eigen::Dynamic, 1 > &grad, Eigen::Matrix< double, Eigen::Dynamic, Eigen::Dynamic > &H)

**Definition:** autodiff.hpp:194


---

[ Stan Home Page ]

*© 2011–2014,
Stan Development Team.*
